# Social support and self-compassion mediate the relationship between alexithymia and quality of life in postoperative breast cancer patients: a cross-sectional study

**DOI:** 10.3389/fpsyg.2025.1722515

**Published:** 2025-12-10

**Authors:** Zejuan Liao, Pinjia Hu, Ting Xiao, Huang Tang, Hanmei Deng, Xueqin Hao, Jun Yan

**Affiliations:** 1 School of Nursing, Sun Yat Sen University, Guangzhou, Guangdong, China; 2 Guangdong Women and Children Hospital, Guangzhou, Guangdong, China

**Keywords:** quality of life, alexithymia, social support, self-compassion, breast cancer

## Abstract

**Background:**

In breast cancer patients, alexithymia has been found to correlate with poorer quality of life. While previous research has established a connection between alexithymia and various outcomes, the mediating effect of social support and self-compassion—promoting quality of life—remains largely unexplored, underscoring the need for further investigation in this area.

**Objective:**

To examine the mediating role of social support and self-compassion in the association between alexithymia and quality of life in postoperative breast cancer patients.

**Methods:**

A cross-sectional correlational study was conducted among 324 postoperative breast cancer patients from a tertiary Grade A hospital in Guangzhou, China. Variables were measured using the Functional Assessment of Cancer Therapy-Breast version 4.0 (FACT-Bv4.0), Toronto Alexithymia Scale (TAS-20), Social Support Rating Scale (SSRS), and Self-Compassion Scale (SCS). Data analyses were performed using descriptive analysis, independent sample t-tests, one-way ANOVA, Pearson correlation analysis, and mediation analyses performed with Hayes’ PROCESS macro for SPSS.

**Results:**

The study identified alexithymia was negatively associated with quality of life. Additionally, social support and self-compassion mediated the relationship between alexithymia and quality of life in postoperative breast cancer patients.

**Conclusion:**

The study highlights the complex interplay between alexithymia, quality of life, social support and self-compassion, emphasizing the significant mediating effects of social support and self-compassion among breast cancer patients. Additionally, the findings imply that interventions targeted at enhancing social support and self-compassion could manage the consequences of alexithymia, and ultimately improve their quality of life.

## 1 Introduction

Breast cancer (BC) remains the predominant malignancy among women on a global scale, and is marked by the first-highest morbidity, mortality, and rejuvenation (Bray et al., 2024). Up to date, surgery is the most predominant treatment for BC (Li et al., 2024). However, the loss of breasts during surgery leads to physical deformities and alters women’s self-perception, causing significant harm to their self-esteem (Collins et al., 2011; Gu et al., 2018). Furthermore, a significant proportion of women experience surgery-induced adverse effects, such as pain, restricted mobility, weakness, fatigue, treatment-related financial toxicity (FT) and changes in interpersonal relationships after surgery (Min et al., 2024; Wang Y. et al., 2024). BC patients frequently develop maladaptive affective responses to these challenges, manifesting as anxiety, depression, fear of recurrence, and other negative emotions, collectively undermining their quality of life (QoL) (Wang X. et al., 2024; Xiong et al., 2025).

Quality of life (QoL) describes an individual’s perceived life position within their cultural context and values, shaped by personal goals, expectations, standards, and concerns (Borsati et al., 2024). QoL assessment has emerged as a pivotal focus area in modern healthcare system evaluations serving as a key comprehensive indicator for monitoring treatment effectiveness and rehabilitation outcomes in BC management (Al-Sharman et al., 2024; Kastora et al., 2023). An expanding body of literature has demonstrated diminished QoL in BC patients following surgical interventions (Abdo et al., 2023; Adam et al., 2023; Faroughi et al., 2023; Jing et al., 2023; Mokhtari-Hessari and Montazeri, 2020; Tommasi et al., 2022). A study conducted in Northern Iran found that 57.3% of postoperative BC patients reported undesirable QoL (Zolfaghary et al., 2023). Therefore, it is vital to both holistically assess QoL determinants and develop tailored interventions. This approach ensures postoperative BC patients continue to have access to readily available support for maintaining a favorable QoL.

### 1.1 Alexithymia and QoL

Theories of stress and coping, self - regulation, personality, and social processes have identified personality attributes, social support, and coping as proximal determinants of health outcomes in cancer patients (Stanton et al., 2007), thereby playing a pivotal role in their QoL (Durá-Ferrandis et al., 2017). The first determinant of QoL is alexithymia, a personality trait characterized by barriers to identifying, describing, and expressing emotions, accompanied by an externally oriented thinking style, restricted imaginative processes, and an inability to differentiate between feelings and bodily sensations of emotional arousal (Taylor and Bagby, 2013). Based on Emotion Regulation Theory (Gross, 1998), individuals with alexithymia face significant limitations in regulating their emotions and are more likely to adopt maladaptive strategies, causing increased physiological stress and depletion of cognitive resources that ultimately result in physical and mental exhaustion as well as functional decline. Concurrently, alexithymia is associated with psychosocial distress and impaired interpersonal functioning, collectively contributing to compromised QoL (Klietz et al., 2020). Empirical evidence supports a strong negative correlation between alexithymia and QoL in patients with BC (Yeung et al., 2020), implying that elevated alexithymia serves as an independent determinant predicting poorer QoL (Alvarado-Bolaños et al., 2023; Culicetto et al., 2024). Furthermore, a study of 119 postoperative BC patients revealed that the prevalence of alexithymia was as high as 33% (Gutiérrez Hermoso et al., 2020). This indicates that alexithymia has become a widespread issue in the domain of BC, necessitating worldwide attention from nursing professionals. Therefore, it is essential for nurses and healthcare professionals to understand the mechanism underlying the association between alexithymia and QoL. In accordance with the above content, the following hypothesis is proposed:

*H1*: Alexithymia is negatively associated with QoL of postoperative BC patients.

### 1.2 The mediating role of social support

Social support—defined as a critical psychosocial resource encompassing material and emotional assistance (Gottlieb and Bergen, 2010)—constitutes the second determinant of QoL, as it reduces the impact of stress on individuals, thereby improving their well-being and health (Cohen and Wills, 1985). Experimental studies demonstrate that social support functions as a critical determinant of QoL in BC patients, with enhanced social support directly contributing to improved QoL outcomes (Yan et al., 2016). In addition, the characterization of alexithymia and its relationship with social support is particularly noteworthy. Specifically, alexithymia can lead to social isolation, a restricted lifestyle, and delayed help-seeking, fostering superficial relationships and conflict avoidance, which, in turn, not only exacerbates social anxiety but also severely impairs their capacity to utilize social support (Karakas, 2016; Lin et al., 2023). Empirically, research indicates that alexithymia negatively predicts social support levels, with heightened alexithymia correlating significantly with diminished social support (Celik et al., 2022). Thus, it can be deduced that the greater the degree of alexithymia, the lower the level of social support received, and consequently leading to a decrease in QoL (Nekouei et al., 2014). In other words, it can be inferred that social support may mediate the relationship between alexithymia and QoL. Notably, whether there is such a mediating mechanism among these three variables for BC patients remains unexplored. Accordingly, the following hypothesis is advanced:

*H2*: Social support plays a mediating role in the influence of alexithymia toward QoL of postoperative BC patients.

### 1.3 The mediating role of self-compassion

Self-compassion—defined by Neff (2003a) as a positive and effective coping style that enables individuals to mitigate perceived threats, navigate challenges, and gain a sense of control over their circumstances (Pinto-Gouveia et al., 2014)—represents the third key determinant of QoL and has been shown to significantly enhance it. Individuals with self-compassion typically approach challenging situations with a balanced perspective rooted in positivity and self-acceptance (Sirois et al., 2015). Thus, self-compassion plays a crucial role in assessing QoL in contexts of cancer care. A study demonstrated that cancer patients with high levels of self-compassion exhibited higher QoL (Garcia et al., 2021). Meanwhile, according to Emotion Exchange Theory (Floyd, 2006), the cognitive and emotional deficits associated with alexithymia hinder intimate and effective emotional communication—both with oneself and others (Ozonder Unal and Ordu, 2023). As a result, this impairment contributes to empathy deficits and a sense of personal experience disconnection from others, leaving individuals with limited internal resources to develop and foster self-compassion (Lyvers et al., 2020). Notably, this phenomenon may be particularly pronounced in China, with Confucian values emphasizing self-sacrifice and relational harmony (Zhang et al., 2025) creating an inherent conflict that further suppresses self-compassion. Consistently, empirical studies have found that reducing alexithymia can lead to an increase in self-compassion (Xu et al., 2024; Zhang et al., 2024). Thus, it is reasonable to infer that patients with higher levels of alexithymia may exhibit lower self-compassion, which in turn negatively impacts their QoL. Similarly, the mediating mechanism through which self-compassion influences the relationship between alexithymia and QoL remains uncertain for BC patients. Hence, on the basis of the above discussion, the study hypothesizes that:

*H3*: Self-compassion plays a mediating role in the influence of alexithymia toward QoL of postoperative BC patients.

### 1.4 The serial-multiple mediating role of social support and self-compassion

It is worth mentioning that self-compassion can be improved by social support. From the perspective of the Thriving through Relationships Theory (Feeney and Collins, 2015), social support functions as an interpersonal process that promotes thriving in both challenging and positive circumstances, enabling recipients to experience feelings of positive self-regard, self-acceptance, calmness, and security, thereby fostering self-kindness and ultimately enhancing their self-compassion. Beyond this, through sustained social support, individuals internalize patterns of kindness and acceptance, which facilitates the redirection of compassion toward themselves, thereby fostering greater self-compassion (Tannous-Haddad et al., 2024). Moreover, in China’s Confucian culture where self-compassion is often other-focused (Tiwald, 2011), empathetic and other-oriented social support fosters a sense of being understood and accepted, which is subsequently transformed into self-kindness, enabling individuals to respond to personal failures with self-compassion (Zhao et al., 2023). Empirical studies have also shown that patients with BC survivors and greater social support have been reported to have significantly higher levels of self-compassion (Masoumi et al., 2022). That is to say, the higher the level of social support, the stronger the ability of self-compassion (Zhou et al., 2022). A study has also revealed that there is a positive correlation between social support and self-compassion (Jeon et al., 2016). Thus, the present study proposes the following hypothesis.

*H4*: Social support and self-compassion play a serial-multiple mediating role between alexithymia and QoL of postoperative BC patients.

**Figure 1:**
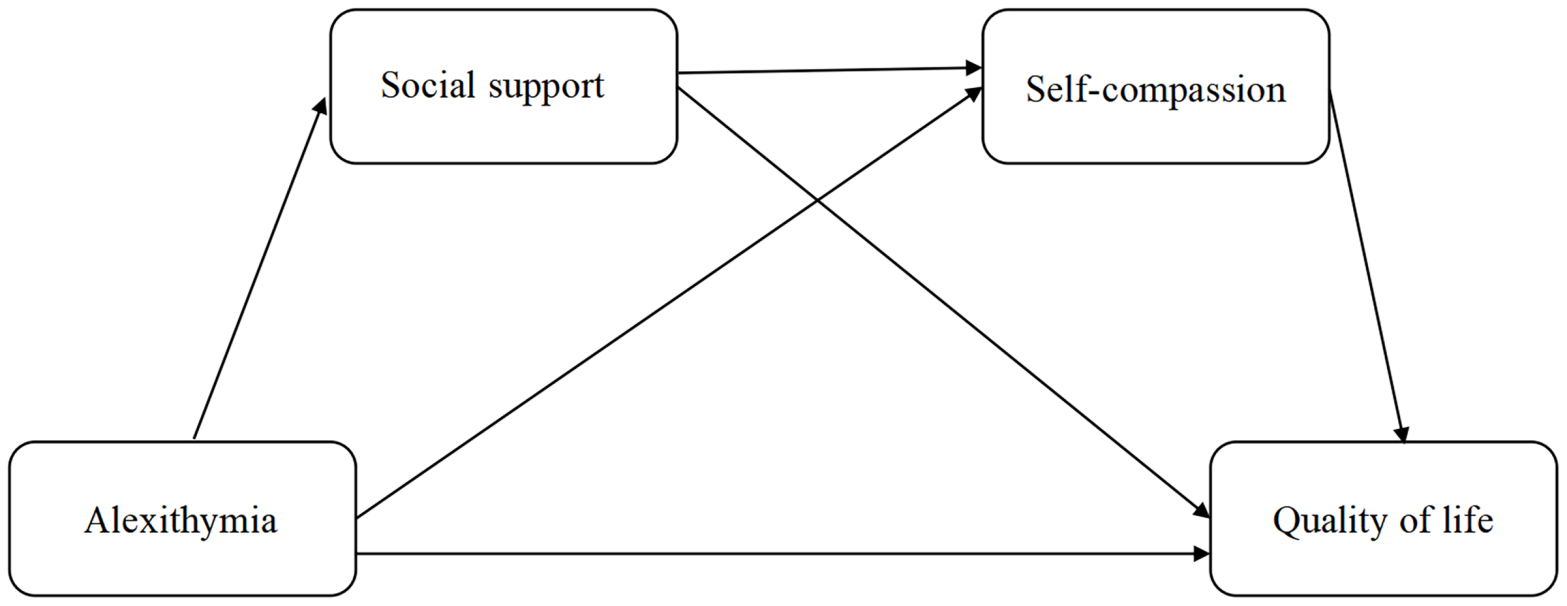
Hypothesized associations between alexithymia, social support, self-compassion, and quality of life (QoL).

In general, there is a gap in knowledge regarding the overall effects of alexithymia, social support, self-compassion on QoL. More importantly, the possible mediating mechanisms involved have not yet been empirically tested. Therefore, the present study aims to address these gaps by examining a serial-multiple mediation model involving these variables. The hypothesized model is presented in Figure. 1.

## 2 Methods

### 2.1 Study design

A cross-sectional correlational design was employed for this research.

### 2.2 Participants and setting

BC patients were recruited by convenience sampling from December 2024 to June 2025 at a tertiary Grade A hospital in Guangzhou, China. Patients fulfilling the subsequent criteria were qualified to take part in this study: (1) diagnosed primary breast cancer by pathological examination; (2) had been receiving surgery for more than 3 months; (3) capable of communicating effectively and finishing the questionnaires; (4) aware of their illness and consented to participate in the study. Patients were excluded based on the following criteria: (1) had been diagnosed with conditions such as depression, schizophrenia, anxiety, dementia, or other mental disorders; (2) co-existence of other grave physical diseases, like severe dysfunction of the kidneys, heart, liver, and other organs; (3) simultaneous presence of malignant neoplasms.

G*Power 3.1.9.2 software was utilized to estimate the sample size. This estimation was grounded in a research framework featuring three predictors of QoL (alexithymia, social support, and self-compassion) and fifteen covariates that captured sociodemographic and clinical characteristics. A two-tailed F-test for multiple linear regression was conducted, assuming an effect size (f^2^) of 0.15, a significance level (*α*) of 0.05, and a statistical power of 0.80. Based on these parameters, the required minimum sample size was determined to be 150. Taking into account a 20% attrition rate, the final minimum sample size was established as 188. The actual sample (*N* = 324) exceeded this minimum, ensuring sufficient statistical power.

### 2.3 Measures

This study utilized self-administered questionnaires to collect sociodemographic and clinical data, supplemented by reliable instruments specifically calibrated for Chinese patient populations. These tools reliably assessed core outcome variables including QoL, alexithymia, social support, and self-compassion, ensuring methodological rigor through established reliability and validity metrics.

#### 2.3.1 Sociodemographic and clinical characteristics

The sociodemographic and clinical characteristics questionnaire was developed and administered, with approval from an expert panel. The sociodemographic survey gathered vital information, including age, body mass index (BMI), marital status, number of children, childbearing needs, level of education, employment status, family per capita monthly income (CNY), medical payment method, habitation, living alone or not, and religious affiliation. Clinical information included TNM staging of cancer, number of combined chronic diseases, and duration of diagnosis of breast cancer (years).

#### 2.3.2 QoL

Participants’ QoL was measured with a Chinese version of the 36-item Functional Assessment of Cancer Therapy-Breast version 4.0 (FACT-Bv4.0) (Wan et al., 2007). The scale measures five domains of QoL: physical well-being (PWB, 7 items), social/family well-being (SWB, 7 items), emotional wellbeing (EWB, 6 items), functional well-being (FWB, 7 items), and breast cancer-specifc (BCS concerns, 9 items). Each item is scored on a 5-point Likert scale (0 = not at all, 4 = very much). Scores range from 0 to 144; higher scores indicate a greater level of QoL (Brady et al., 1997). The scale demonstrates satisfactory validity and good reliability, with Cronbach’s *α* for the five subscales ranging from 0.59 to 0.85. In this study, Cronbach’s *α* was 0.92.

#### 2.3.3 Alexithymia

Alexithymia was assessed using the 20-Item Toronto Alexithymia Scale (TAS-20) developed by Bagby et al. (1994), which has demonstrated widespread adoption for estimating patients’ trait alexithymia. The scale includes three factors associated with alexithymia: difficulty in identifying feelings (DIF, 7 items), difficulty in describing feelings (DDF, 5 items) and externally oriented thinking (EOT, 8 items). The TAS-20 is scored on a 5-point Likert scale ranging from “strongly disagree” (1) to “strongly agree” (5). Total scores range from 20 (minimum) to 100 (maximum); higher scores indicate a greater level of alexithymia. A cutoff score of ≥ 61 is considered a significant level of alexithymia. The questionnaire exhibited strong internal consistency and temporal stability, with its three-factor structure demonstrating theoretical alignment with alexithymia’s core dimensions. In the present study, Cronbach’s *α* for the total score was 0.87.

#### 2.3.4 Social support

Social support was assessed with the Social Support Rating Scale (SSRS) developed by Xiao (1994), which includes 10 items across three domains (subjective support, objective support, and support utilization). Items are scored from 1 to 4 points, resulting in a total score ranging from 12 to 66, where higher scores indicate better perceived social support. The total score can be classified into 3 categories: low levels (12–22), moderate levels (23–44), and high levels (45–66) of social support. The SSRS exhibited excellent internal consistency (Cronbach’s *α* = 0.92) (Xiao, 1994), and it demonstrated good internal consistency in the current study (Cronbach’s *α* = 0.79).

#### 2.3.5 Self-compassion

The 26-item Self-Compassion Scale (SCS), originally developed by Neff (2003b), was employed to assess an individual’s level of self-compassion. The scale encompasses six subscales: three measuring positive attributes (self-kindness, common humanity and mindfulness) and three assessing negative tendencies (self-judgement, isolation and over-identified). Participants rated their responses on a 5-point Likert scale, ranging from “never” (1) to “always” (5), with negative composite items scored in reverse. Total scores range from 26 to 130. The higher the score, the greater the self-compassion. The scale demonstrated exceptional internal consistency (Cronbach’s *α* = 0.92). In this study, the Cronbach’s *α* coefficient was 0.90.

### 2.4 Data collection

For patients who consented to participate, the questionnaires were available for completion either during a clinic visit or at a time that was convenient for the participant. Participants received a document package including sociodemographic and clinical surveys, as well as self-report questionnaires. For those unable to complete the questionnaires in writing, the lead author read each question aloud and documented the responses. The time required to finish the questionnaires ranged from approximately 15 to 20 min per participant.

### 2.5 Ethical consideration

Ethical approval for this study was granted by Guangdong Women and Children Hospital Medical Ethics Committee (Registration number: 202401377). For patients meeting the eligibility criteria, the lead author delivered both oral and written details regarding the study procedures. Patients received information that their participation was optional, with clear communication that the choice to participate or withdraw at any point would not impact their medical care. Participants were additionally assured that their data would remain anonymous and be safeguarded. All patients gave written informed consent to participate prior to data collection.

### 2.6 Statistical analysis

Data analysis was performed using IBM SPSS Statistics Version 26. Descriptive statistical methods were employed to characterize the sociodemographic and clinical attributes of participants. For continuous variables, results were reported as mean and standard deviation (SD), while categorical variables were described using frequency counts and percentages.

The relationships among alexithymia, social support, self-compassion, and QoL were evaluated using Pearson’s bivariate correlation to examine associations between scale scores. Before conducting model testing, t-tests and one-way analysis of variance (ANOVA) were conducted to examine the associations between QoL and sociodemographic/clinical characteristics, aiming to identify any covariates requiring control in the subsequent analysis. For the serial-multiple mediation hypotheses, Model 6 of the PROCESS 4.2 macro, developed by Hayes (2022), was used to conduct mediation analysis with 95% bias-corrected confidence intervals (CI) using 5,000 bootstrap samples. If the 95% CI for the estimate does not contain zero, the mediating effect is confirmed significant. A two-tailed *p*-value < 0.05 was defined as statistically significant.

## 3 Results

### 3.1 Data quality control and statistical assumptions

Rigorous quality control measures were implemented throughout the data collection and analysis stages. During data collection, a total of 330 questionnaires were distributed. Among them, one participant refused to participate. Additionally, five questionnaires were considered invalid due to omission of 10% or more of the answers, and obvious logical errors in responses. The remaining 324 valid questionnaires were then entered into a database independently by two research assistants, and all entries were cross-verified to ensure data accuracy. Consequently, this final dataset of 324 questionnaires was included for analysis, resulting in a high response rate of 98.2%.

Prior to conducting parametric analyses, we further verified that all necessary statistical assumptions were met. Normality was confirmed based on acceptable skewness and kurtosis values, supported by visual examination of the Quantile-Quantile (Q-Q) plots. Multicollinearity was assessed using the variance inflation factor (VIF), with values ranging from 1.251 to 1.451, indicating no concerning multicollinearity. The Durbin-Watson statistic was 1.755, supporting the independence of residuals. Homoscedasticity was evaluated by visually inspecting the scatter plot of regression-standardized residuals against regression-standardized predicted values, which revealed a relatively even distribution.

### 3.2 Descriptive statistics and univariate analysis

Table 1 shows participants’ sociodemographic and clinical characteristics. A total of 324 women with BC were enrolled in the study. The majority of participants were aged between 36 and 50 years (49.7%), and reported a monthly income of 3,000–5,999 yuan (equivalent to US$418–835; 38.3%). Predominantly, participants had one or more children (95.4%), no childbearing needs (92.0%), no religious affiliation (92.3%), and were married (87.7%). Additionally, 84.6% had attained junior high school education or higher, 64.8% were employed, 98.5% had medical insurance. Regarding living conditions, 76.5% resided in urban areas, and 91.7% lived with their families. Clinically, 84.6% suffered no combined chronic diseases, 86.4% had a BC duration of less than 5 years, and 78.7% fell TNM II stage or lower.

**Table 1:** Descriptive statistics and related to quality of life in breast cancer patients.

Variables	*n* (%)	QoL (M ± SD)	*F/t*	*p*
Age, years			1.297	0.275
21–35	34 (10.5)	99.65 ± 23.94		
36–50	161 (49.7)	105.99 ± 20.57		
≥51	129 (39.8)	104.56 ± 20.61		
BMI			0.271	0.763
<18.5	14 (4.3)	102.79 ± 24.27		
18.5 ~ 24.9	218 (67.3)	105.34 ± 20.76		
≥25	92 (28.4)	103.66 ± 21.16		
Marital status			−1.162	0.246
Single/divorced/widowed	40 (12.3)	101.15 ± 21.55		
Married	284 (87.7)	105.26 ± 20.88		
Number of children			2.085	0.102
0	15 (4.6)	103.00 ± 20.19		
1	112 (34.6)	107.81 ± 20.45		
2	154 (47.5)	104.42 ± 20.72		
≥3	43 (13.3)	98.63 ± 22.67		
Childbearing needs			6.047	0.003
No	298 (92.0)	105.75 ± 20.33		
Yes	14 (4.3)	100.43 ± 22.41		
Uncertain	12 (3.7)	85.17 ± 26.47		
Level of education			8.201	<0.001
Primary and below	50 (15.4)	97.10 ± 19.86		
Junior high school	89 (27.5)	99.03 ± 22.22		
Senior high school/technical secondary	71 (21.9)	109.66 ± 19.94		
College and above	114 (35.2)	109.53 ± 19.14		
Employment status			6.198	0.002
Employed/Part-time	210 (64.8)	106.02 ± 21.45		
Retired	76 (23.5)	106.79 ± 19.09		
Unemployed	38 (11.7)	93.71 ± 19.03		
Family per capita monthly income (CNY)			4.705	0.003
<3,000	101 (31.2)	99.20 ± 22.13		
3,000 ~ 5,999	124 (38.3)	105.03 ± 20.58		
6,000 ~ 8,999	59 (18.2)	110.76 ± 19.08		
≥9,000	40 (12.3)	109.08 ± 18.89		
Medical payment method			−0.382	0.703
Self-pay	5 (1.5)	101.20 ± 23.74		
Insurance	319 (98.5)	104.81 ± 20.97		
Habitation			1.588	0.113
Urban	248 (76.5)	105.78 ± 20.49		
Rural	76 (23.5)	101.42 ± 22.31		
Living alone or not			0.063	0.950
Yes	27(8.3)	105.00 ± 23.11		
No	297(91.7)	104.73 ± 20.82		
Religious affiliation			2.490	0.013
No	299 (92.3)	105.59 ± 20.64		
Yes	25 (7.7)	94.80 ± 22.86		
TNM			2.475	0.061
0	13 (4.0)	114.77 ± 11.11		
I	79 (24.4)	106.61 ± 20.71		
II	163 (50.3)	105.12 ± 21.69		
III	69 (21.3)	99.90 ± 20.21		
Number of combined chronic diseases			0.878	0.381
None	274 (84.6)	105.19 ± 21.33		
≥ 1	50 (15.4)	102.36 ± 18.93		
Duration of diagnosis of BC (years)			9.694	<0.001
≥ 3 months to < 1	136 (42.0)	98.91 ± 21.26		
1–5	144 (44.4)	109.37 ± 19.50		
>5	44 (13.6)	107.73 ± 20.82		

QoL, quality of life; BC, breast cancer; M, Mean; SD, Standard deviation; CNY, China Yuan.

Table 1 also summarizes the results of the univariate analyses regarding quality of life across various sociodemographic and clinical factors. The results revealed statistically significant differences between groups based on childbearing needs, level of education, employment status, family per capita monthly income, religious affiliation, and duration of diagnosis of breast cancer.

### 3.3 Descriptive statistics and correlation analyses

Table 2 presents the descriptive statistics and bivariate correlations for the study variables. Pearson correlation analysis revealed there were significant relationships among variables. Specifically, alexithymia was significantly negatively correlated with social support, self-compassion, and QoL. In contrast, social support was significantly positively correlated with self-compassion and QoL. Additionally, self-compassion was positively and significantly correlated with QoL.

**Table 2:** Descriptive statistics for outcome variables and Pearson’s bivariate correlation.

Variable	Range	M ± SD	1	2	3	4
1. Alexithymia	20–100	56.49 ± 12.36	1.000			
2. Quality of life	0–144	104.76 ± 20.98	−0.528^**^	1.000		
3. Social support	12–66	41.33 ± 7.81	−0.369^**^	0.493^**^	1.000	
4. Self-compassion	26–130	95.30 ± 14.91	−0.506^**^	0.627^**^	0.405^**^	1.000

^**^p < 0.01; M, Mean; SD, Standard deviation.

### 3.4 Multiple mediator analysis for the study variable

Prior to mediation analysis, covariates were selected and controlled based on statistically significant associations identified in univariate analyses. The study examined a mediation model with alexithymia as the independent variable, social support and self-compassion as mediators, and QoL as the dependent variable. Both unstandardized (B) and standardized (*β*) coefficients are reported in Tables 3, 4 for better interpretability and comparability, with the significance of the mediating effects determined by the bootstrap confidence intervals for the unstandardized coefficients.

**Table 3:** Regression equation for mediation analysis between alexithymia, social support, self-compassion and quality of life in breast cancer patients.

**Outcome variable**	**Predictor variable**	***R***^***2***^	***F***	***B***	***SE***	***β***	***t***	**LLCI**	**ULCI**
Social support	Alexithymia	0.162	7.594^***^	−0.204	0.037	−0.323	−5.548^***^	−0.277	−0.132
Self-compassion		0.348	18.651^***^						
	Alexithymia			−0.523	0.065	−0.434	−8.047^***^	−0.651	−0.395
	Social support			0.483	0.095	0.253	5.085^***^	0.296	0.670
Quality of life		0.525	34.660^***^						
	Alexithymia			−0.369	0.086	−0.217	−4.295^***^	−0.538	−0.200
	Social support			0.619	0.119	0.231	5.210^***^	0.385	0.853
	Self-compassion			0.549	0.068	0.390	8.091^***^	0.415	0.682
Quality of life	Alexithymia	0.335	19.865^***^	−0.837	0.088	−0.493	−9.504^***^	−1.010	−0.663

***p < 0.001; *В*, unstandardized coefficients; *β*, standardized coefficients; SE, Standard Error; LLCI, Lower Limit Confidence Interval; ULCI, Upper Limit Confidence Interval. 95% CIs (5,000 samples) of the unstandardized coefficients (LLCI/ULCI) were used to test indirect effects (significant if CI excludes zero). Covariates include childbearing needs, level of education, employment status, family per capita monthly income, religious affiliation, TNM staging of cancer, duration of diagnosis of breast cancer.

**Table 4:** Final mediation model for total, direct, and indirect effects between the outcome variables.

Path	Effect	*β*	BootSE	LLCI	ULCI
Total effect	−0.837^***^	−0.493^*^	0.088	−1.010	−0.663
Direct effect					
Alexithymia → quality of life	−0.369^***^	−0.217^*^	0.086	−0.538	−0.200
Total indirect effect	−0.468^*^	−0.276^*^	0.070	−0.614	−0.336
Indirect 1: alexithymia → social support →quality of life	−0.126^*^	−0.074^*^	0.035	−0.201	−0.065
Indirect 2: alexithymia → self-compassion →quality of life	−0.287^*^	−0.169^*^	0.055	−0.402	−0.188
Indirect 3: alexithymia → social support→ self-compassion →quality of life	−0.054^*^	−0.032^*^	0.017	−0.091	−0.025

***p < 0.001, *p < 0.05; *β*, standardized coefficients; BootSE, Bootstrap Standard Error; LLCI, Lower Limit Confidence Interval; ULCI, Upper Limit Confidence Interval. 95% CIs (5,000 samples) of the unstandardized coefficients (LLCI/ULCI) were used to test indirect effects (significant if CI excludes zero).

The specific results of the regression analysis are presented in Table 3. All regression coefficients showed statistically significant results (*p* < 0.001). Alexithymia negatively predicted social support, self-compassion, and QoL. Meanwhile, social support positively predicted self-compassion and QoL, and self-compassion positively predicted QoL.

**Figure 2:**
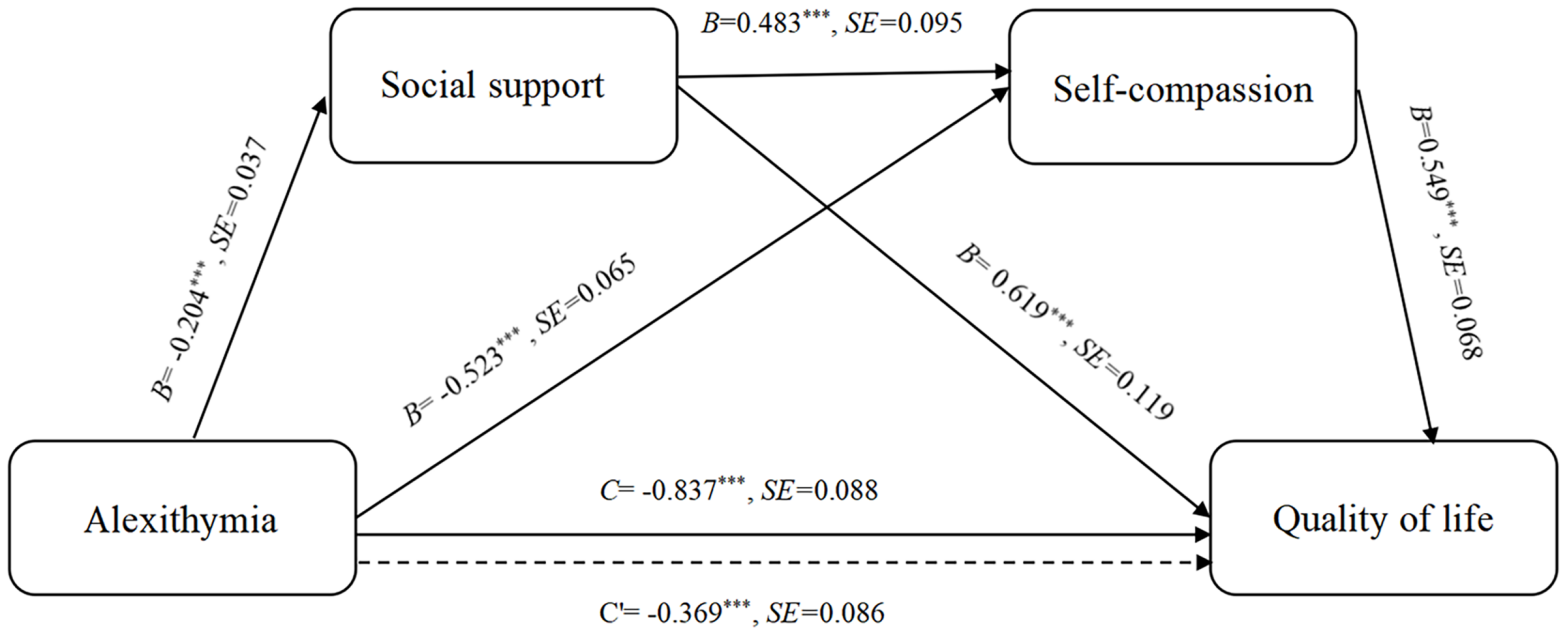
Serial-multiple mediation of social support and self-compassion in the relationship between alexithymia and QoL with non-standardized beta values and standard error. ***p < 0.001, *B*, unstandardized coefficients; SE, Standard Error; C, total effect; C', direct effect.

The mediation was tested using the non-parametric Bootstrap method with 5,000 samples. The results showed that the confidence interval for the direct effect of alexithymia on QoL did not include zero, which verifies that alexithymia has a significant direct effect on QoL, with a direct effect value of −0.369. Additionally, the bootstrap 95% confidence intervals for the mediating effects of social support and self-compassion also did not contain zero, indicating that both variables function as mediators in the relationship between alexithymia and QoL. Specifically, the mediating effects of social support and self-compassion consisted of three paths, with respective effect values of −0.126 for Path 1, −0.287 for Path 2, and −0.054 for Path 3. In this study, the total indirect effect was −0.468, accounting for 55.91% of the total effect of −0.837. Detailed results are presented in Table 4 and illustrated in Figure 2. Overall, these results demonstrated a serial-multiple mediation model.

## 4 Discussion

The primary aim of our study was to examine the roles of social support and self-compassion in mediating the relationship between alexithymia and QoL among postoperative BC patients in China. Our findings demonstrated that alexithymia exerted a significant negative effect on QoL. Moreover, social support and self-compassion not only partially mediated this relationship but also played a serial-multiple mediating role, highlighting the complex interconnections among these variables.

As a vital characteristic personality in postoperative BC patients, alexithymia distorts body image, compromises disease adaptation, and diminishes QoL (Gutiérrez Hermoso et al., 2020). The existing literature supports that alexithymia—a factor integral to emotion somatization, social dysfunction, and psychological distress—is linked to decreased QoL when elevated (Culicetto et al., 2024; Gutiérrez-Hermoso et al., 2022; Renzi et al., 2020). Our findings add to this mounting evidence, emphasizing the importance of alexithymia in somatic health, psychological well-being, and QoL. While alexithymia, as a personality trait, is relatively stable and difficult to change, research suggests that the emotional difficulties associated with it can be effectively alleviated (Pierro et al., 2022). This underscores the importance of continuous research to identify factors that can reduce alexithymia levels, thereby contributing to improved physical and mental health as well as QoL in postoperative BC patients.

Our study demonstrated that social support served as a significant mediator in the relationship between alexithymia and QoL. This finding is consistent with previous research that social support as a pivotal factor in ameliorating alexithymia and glycaemic control among diabetes patients (Celik et al., 2022). Social support contributes to emotional regulation and cancer-related stress recovery in postoperative BC patients, thereby improving psychological well-being and health outcomes, and ultimately mitigating impaired QoL due to alexithymia (Cho et al., 2024; Pei et al., 2021). These findings suggest that healthcare providers wishing to alleviate alexithymia and enhance QoL among postoperative BC patients should prioritize initiatives that strengthen social support networks (Zhu et al., 2023). For this purpose, designing social support intervention programs represents an effective strategy (Ban et al., 2021). For example, Liu et al. (2024) developed a social support intervention model for postoperative BC patients, which primarily focused on encouraging emotional expression while fostering the development of adaptive coping strategies, ensuring patients receive comprehensive support encompassing physical and emotional well-being, ultimately improving their QoL. By addressing social support, healthcare providers can foster a supportive care environment that alleviates alexithymia and enhances QoL.

Building upon the importance of social support, self-compassion functioned as a protective mediator, mitigating the adverse effects of alexithymia on QoL. This conclusion aligns with the prior study identifying self-compassion acts as a crucial mediator in the relationship between alexithymia and postpartum depression (Ana-Maria et al., 2023), suggesting a parallel buffering mechanism across different health outcomes. Self-compassion serves as a crucial inner resource for cancer patients (Wang et al., 2025), which can help them treat themselves with acceptance and care and view cancer as part of the common human experience, as well as to enable them to confront challenges with greater calmness, objectivity, and positivity (Neff, 2003a), thus empowing patients to face cancer-related physical and psychological adversities (Dryer et al., 2022; Przezdziecki et al., 2013; Treaster, 2018), and contributing to offset the impact of alexithymia on QoL. Therefore, future research should integrate self-compassion as a core component of QoL interventions for BC patients. On the one hand, healthcare workers are encouraged to actively explore and master strategies and methods for fostering self-compassion. On the other hand, medical staff should also establish supportive conditions to effectively enhance self-compassion, such as mindfulness-based self-compassion interventions (Kılıç et al., 2021) and cognitively-based compassion training (Gonzalez-Hernandez et al., 2018). These results suggest healthcare staff can enhance QoL by implementing self-compassion programs that cultivate this capacity as a protective component, resulting in reduced alexithymia.

Building on our exploration, we next found that alexithymia was able to impact QoL in postoperative BC patients through a serial-multiple mediation of social support and self-compassion. This elucidates the key mechanism linking these variables to QoL outcomes in this population. Our findings indicate that social support significantly and positively influences self-compassion, which aligns with previous literature showing that social support enhances life satisfaction in infertile women by boosting their self-compassion (Chu et al., 2021). Acting as a “safe haven,” social support creates a supportive environment that fosters feelings of kindness and care (Feeney and Collins, 2015; Liu et al., 2021), through which patients learn to extend similar caring attitudes toward themselves (Tannous-Haddad et al., 2024), thus elevating self-compassion and ultimately promoting long-term well-being and enhanced QoL. Empirical support comes from a study conducted by Brooks et al. (2022), which demonstrated that individuals reporting stronger social support exhibited higher self-compassion, resulting in higher mental health-related quality of life. This highlights the value of interventions that target social support and self-compassion to enhance QoL. For instance, the Mindful Self-Compassion (MSC) program, developed by Neff and Germer (2013) and delivered through peer support groups, not only fosters a robust social support network but also enhances self-compassion, thereby improving happiness, life satisfaction, and overall QoL. In conclusion, this study suggests that while modifying alexithymia as a personality trait proves difficult, social support and self-compassion represent malleable factors. These can be enhanced therapeutically through individual-level approaches to improve QoL in postoperative BC patients.

## 5 Clinical implications

Our study highlights key practical implications. First, it underscores the critical impact of alexithymia, social support, and self-compassion on postoperative BC patients’ QoL, advocating for prioritized screening of alexithymia in high-risk individuals. This approach may enable more comprehensive care and potentially prevent QoL deterioration. Second, our findings reveal that social support and self-compassion play significant serial-multiple mediating roles in the relationship between alexithymia and QoL. Understanding these mechanisms provides valuable insights for developing targeted interventions. Consequently, future research should prioritize culturally adapted interventions focusing on social support and self-compassion. This is particularly urgent in Chinese contexts, where Confucian ethics emphasizing others’ needs often obscure the health impact of self-compassion (Chien, 2016). Localized optimization of intervention designs is therefore essential to ensure relevance and efficacy.

## 6 Limitations and future directions

Although our study offers important insights, it does have some limitations. First, the study’s cross-sectional design constrained our ability to identify causal relationships among the variables. Future studies should duplicate and expand our results using longitudinal or experimental research methods. Second, as a quantitative survey, this study may miss some nuances that a mixed-methods approach could have provided. Third, the scales and measurements employed might not entirely prevent subjective reporting distortion, with the possibility of recall bias or other errors. Lastly, the results of this single-country study may not be entirely generalizable to other cultural or healthcare contexts, which supports the need for additional comparative studies. Therefore, future research overcoming these limitations will contribute to refining our comprehension of alexithymia and and QoL within the clinical practice.

## 7 Conclusion

The present study presented a serial-multiple mediation model, underscoring the complex interplay among alexithymia, social support, self-compassion and QoL in postoperative BC patients. It suggests that multifaceted approaches are required to manage these dynamics. Our results indicate that although altering alexithymia as a personality trait may pose challenges, its negative impact can be positively influenced by factors like social support and self-compassion. Both of the aforementioned significantly boost QoL. Hence, creating a supportive and inclusive relationship and fostering strong self-compassion can be instrumental in mitigating the impact of alexithymia on QoL in postoperative BC patients.

## Data Availability

The raw data supporting the conclusions of this article will be made available by the authors, without undue reservation. Further inquiries can be directed to the corresponding author.
